# Exploring the Genetic Basis of Drought Tolerance in *Alhagi camelorum*: A Comprehensive Transcriptome Study of Osmotic Stress Adaptations

**DOI:** 10.3390/ijms252312725

**Published:** 2024-11-27

**Authors:** Gangliang Tang, Xiangyi Li, Fanjiang Zeng, Junning Ma, Pingyin Guan, Bo Zhang

**Affiliations:** 1 State Key Laboratory of Desert and Oasis Ecology, Xinjiang Institute of Ecology and Geography, Chinese Academy of Sciences, Urumqi 830011, China; tanggangliang@ms.xjb.ac.cn (G.T.); lixy@ms.xjb.ac.cn (X.L.); zengfj@ms.xjb.ac.cn (F.Z.); 2 Xinjiang Key Laboratory of Desert Plant Roots Ecology and Vegetation Restoration, Xinjiang Institute of Ecology and Geography, Chinese Academy of Sciences, Urumqi 830011, China; 3 Cele National Station of Observation and Research for Desert-Grassland Ecosystems, Cele 848300, China; 4 Institute of Horticultural Crops, Xinjiang Academy of Agricultural Sciences, Urumqi 830013, China; junning.ma@hotmail.com; 5 College of Horticulture, China Agricultural University, Beijing 100193, China; 6 National Engineering Technology Research Center for Desert-Oasis Ecological Construction, Xinjiang Institute of Ecology and Geography, Chinese Academy of Sciences, Urumqi 830011, China

**Keywords:** *Alhagi camelorum*, drought resistance, transcriptomic analysis, phytohormones, transcription factors

## Abstract

*Alhagi camelorum*, a desert shrub known for its impressive drought tolerance, exhibits notable resilience under arid conditions. However, the underlying mechanisms driving its drought resistance remain largely unexplored. This study aims to investigate these mechanisms by exposing *A. camelorum* to osmotic stress using varying polyethylene glycol (PEG) concentrations (1%, 5%, 10%) in a controlled laboratory setting. Growth analysis revealed significant inhibition and phenotypic changes with increasing PEG levels. Transcriptomic analysis, including differentially expressed gene identification, GO enrichment analysis, and hierarchical cluster analysis of genes in roots and shoots, identified key pathways associated with drought adaptation, such as ABA-activated signaling, cell wall biogenesis, photosynthesis, and secondary metabolite biosynthesis. Notably, some genes involved in these pathways exhibited tissue-specific expression patterns and showed PEG concentration-dependent regulation. Key findings include the dose-dependent (R^2^ > 0.8) upregulation of a proline-rich protein (Asp01G030840) and a BURP domain-containing protein (Asp02G039780), as well as critical genes involved in cell wall biogenesis (encoding Pectinesterase inhibitor domain-containing and Fasciclin-like arabinogalactan protein), and secondary metabolite biosynthesis (encoding enzymes for terpenoid and flavonoid biosynthesis). The regulation of these genes is likely influenced by phytohormones such as ABA and other stress-related hormones, along with significant transcription factors like ABI4, TALE, MYB61, GRAS, and ERF. These insights lay the groundwork for further research into the functional roles of these genes, their regulatory networks, and their potential applications in enhancing drought resistance in desert plants and agricultural crops.

## 1. Introduction

Camelthorn, belonging to the *Alhagi* genus in the Fabaceae family, is a perennial shrub characterized by its ability to thrive in arid conditions. It is known for its many thorns. The flowers of the camelthorn plant are small and have pinkish peduncles. They are located in the higher regions of the plant. The fruits of the plant range in color from brown to red [1,2]. Camelthorn, a perennial plant, has a broad geographical range and exhibits rapid reproduction. This plant plays a crucial role in safeguarding sandy hills by preventing soil erosion. The herb is highly renowned for its therapeutic attributes and is often used in the management of gastrointestinal diseases, kidney stones, and rheumatic pain [3]. The six most important species of camelthorn are *A. maurorum* (synonymous with *A. persarum*, *A. camelorum*, and *A. pseudalhagi*), *A. canescens*, *A. graecorum* (synonymous with *A. mannifera*), *A. kirghisorum*, *A. nepalensis*, and *A. sparsifolia* [4]. Native to arid and semi-arid regions of Asia and Africa, *A. camelorum* exhibits remarkable resilience to drought and other environmental stresses. Its ability to thrive in harsh conditions makes it an attractive model for studying plant stress responses and adaptation mechanisms [2,5].

The active compounds of *A. camelorum*, for instance, flavonoids, alkaloids, saponins, and tannins, which contribute to its antioxidant, anti-inflammatory, and antimicrobial properties, have been identified in recent years [1,6]. *A. camelorum* has medicinal applications, including promoting wound healing, managing diabetes, and protecting the liver [1]. Ecologically, *A. camelorum* aids in soil stabilization, supports livestock as a forage crop, and thrives in drought and saline conditions [3]. Advances in genetic research and tissue culture have facilitated its propagation and enhanced secondary metabolite production [7]. Overall, *A. camelorum*’s diverse applications and adaptability make it a plant of considerable scientific interest and practical value.

Despite its ecological significance, the intrinsic mechanisms that confer its drought resistance have remained largely enigmatic. Recent pot experiments have unveiled that seedlings of *A. camelorum* exhibit high dry matter storage, robust defense capabilities, and low water consumption under drought stress, suggesting a complex array of adaptive traits [8]. Furthermore, studies by Ullah et al. have demonstrated that the camelthorn *A. sparsifolia* benefits from drought priming, leading to increased root biomass, relative water content in leaves, and enhanced chlorophyll a and carotenoid content under subsequent drought stress conditions [9]. These physiological adaptations are complemented by upregulated activities of antioxidant enzymes such as superoxide dismutase and catalase, as well as the maintenance of the ascorbate-glutathione cycle and related enzyme activities, which are crucial for the regulation of reactive oxygen species. Additionally, the levels of plant hormones like brassinosteroids, jasmonic acid, and abscisic acid are increased, indicating a role in drought stress signaling and adaptation [9]. Nevertheless, the precise molecular underpinnings of the drought adaptation process in *A. camelorum* have yet to be uncovered.

Osmotic stress, a component of drought stress, resulting from water deficit or excess salts in the soil, poses a significant threat to plant growth and productivity in arid regions [10,11]. Polyethylene glycol (PEG) is commonly used to induce osmotic stress in experimental studies due to its ability to reduce water availability to plant roots without introducing ionic toxicity [12,13]. PEG-induced osmotic stress affects water uptake and loss, reduces chlorophyll content, and decreases photosynthetic rates, ultimately inhibiting plant growth [13,14]. Biochemically, plants under PEG stress accumulate proline, aiding in osmotic adjustment and cellular protection. Molecular studies have shown significant gene expression changes, particularly in stress signaling pathways [15]. Antioxidant defenses are also enhanced under PEG treatment, with increased activities of enzymes like superoxide dismutase and catalase, along with higher levels of non-enzymatic antioxidants such as ascorbate and glutathione, which help mitigate oxidative damage [16,17]. Hormonal regulation plays a crucial role, with elevated abscisic acid (ABA) levels modulating stress-responsive genes and interactions with ethylene and jasmonic acid pathways further enhancing stress responses [18,19]. However, the specific genes or pathways that contribute to the PEG-mediated osmotic adaptation in *A. camelorum* remain to be elucidated.

To achieve this, we have designed a comprehensive experimental approach that combines techniques of physiological and transcriptomic analysis in both root and shoot tissues to assess the plant’s response to controlled osmotic stress. Our approach involves subjecting *A. camelorum* to a range of PEG concentrations (1%, 5%, and 10%) in a controlled laboratory setting, which simulates the progressive osmotic stress that plants encounter during water deficit conditions. This allows us to study the tissue-specific and dose-dependent responses of the plant’s molecular and physiological reactions to drought. This is also essential for identifying specific genes and pathways that are upregulated or downregulated in a concentration-dependent manner, providing insights into the plant’s adaptive strategies under varying levels of stress. Through this approach, we seek to uncover the complex molecular mechanisms that drive the plant’s drought tolerance. The findings from this study will not only enhance our understanding of the fundamental biology of drought resistance in desert plants but also offer potential targets for genetic engineering to improve drought tolerance in agricultural crops.

## 2. Results

### 2.1. PEG Treatment Inhibits A. camelorum Seedling Growth

**Figure 1:**
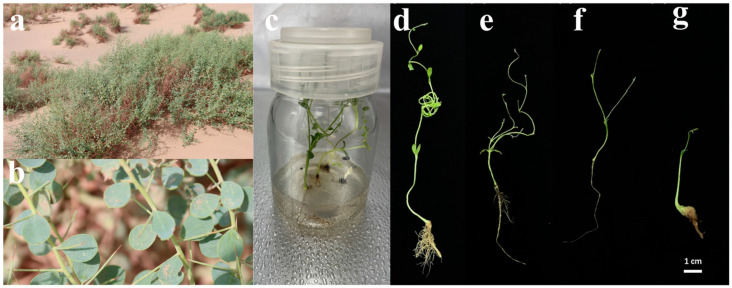
Effects of polyethylene glycol (PEG) on the growth and morphology of *A. camelorum*. (**a**) Representative photograph of *A. camelorum* thriving in its natural desert habitat, showcasing the plant’s adaptation to arid conditions. (**b**) Close-up view of *A. camelorum* in the desert, highlighting the plant’s structural features that enable survival in extreme environments. (**c**) Growth of *A. camelorum* from seeds on Murashige and Skoog (MS) medium within a plant culture bottle, demonstrating the plant’s ability to germinate and grow under controlled conditions. (**d**) Solvent control, illustrating the baseline growth without PEG treatment. (**e**–**g**) Progressive increase in PEG concentration treatments: (**e**) 1% PEG, (**f**) 5% PEG, and (**g**) 10% PEG, showing the differential responses of *A. camelorum* to osmotic stress induced by varying PEG concentrations. Scale bar represents 1 cm.

*A. camelorum* plant exhibited distinct phenotypic alterations in response to PEG-induced osmotic stress. As the concentration of PEG increased, plants showed reduced growth, evidenced by decreased plant height, internode length, as well as inhibited root elongation (Figure 1). The severity of phenotypic changes correlated with the concentration of PEG, with higher concentrations causing more pronounced growth inhibition.

### 2.2. Identification of Differentially Expressed Genes (DEGs)

To further explore the osmotic stress adaptation mechanisms of *A. camelorum*, we conducted transcriptome sequencing on the root and shoot tissues of plants treated with 1%, 5%, and 10% PEG. The first part of our transcriptome analysis focused on the identification of differentially expressed genes (DEGs) in response to PEG treatment in both root and shoot tissues.

**Figure 2:**
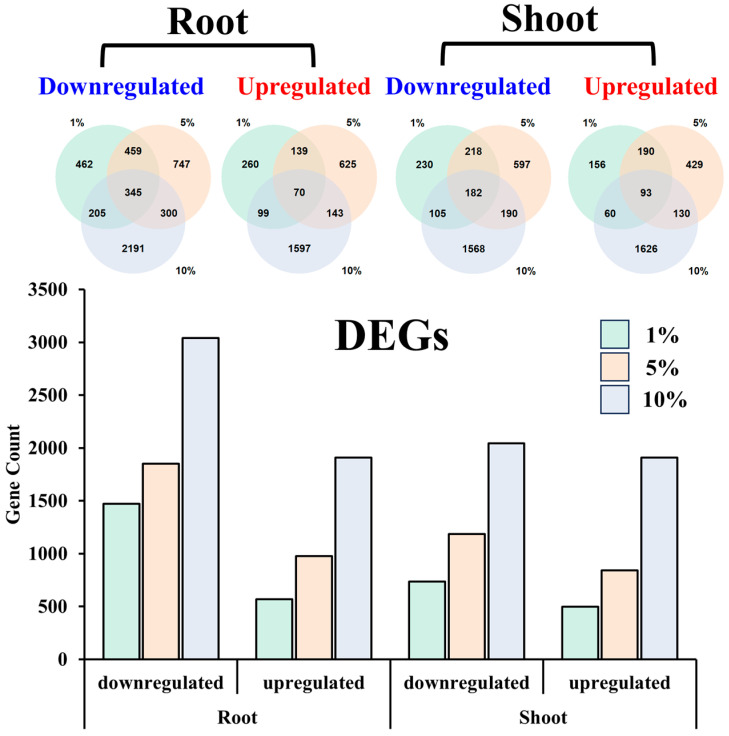
Differentially expressed genes (DEGs) in root and shoot tissues across different PEG concentrations.

Among the 17,159 mapped genes (Table S1), our differential expression analysis revealed a significant increase in the number of DEGs in both root and shoot tissues as the concentration of PEG increased (Figure 2). This trend was consistent for both downregulated and upregulated DEGs. Specifically, in the root tissue treated with 1% PEG, we identified 1471 downregulated genes, which increased to 1851 in the 5% PEG treatment, and further to 3041 in the 10% PEG treatment. Similarly, the number of upregulated genes in the root tissue increased from 568 in the 1% PEG treatment to 977 in the 5% PEG treatment, and to 1909 in the 10% PEG treatment.

Comparatively, in the shoot tissue, the number of downregulated genes was 735 in the 1% PEG treatment, which rose to 977 in the 5% PEG treatment, and to 2045 in the 10% PEG treatment. The upregulation of genes in the shoot tissue also showed a similar pattern, with 499 upregulated genes in the 1% PEG treatment, 842 in the 5% PEG treatment, and 1906 in the 10% PEG treatment.

The analysis of DEGs across different PEG concentrations within the same tissue revealed both shared and unique gene responses (Figure 2). In the root tissue, 345 genes were commonly downregulated across all PEG concentrations, with 462, 747, and 2191 genes being specifically downregulated at 1%, 5%, and 10% PEG, respectively. Similarly, 70 genes were upregulated under all conditions, with 260, 625, and 1597 genes uniquely upregulated at the respective PEG concentrations.

In the shoot tissue, 182 genes were found to be downregulated under all PEG treatments, with an additional 230, 597, and 1568 genes being uniquely downregulated at 1%, 5%, and 10% PEG, respectively. For upregulated genes in the shoot, 93 were common to all treatments, accompanied by 156, 429, and 1626 genes that were specifically upregulated at the different PEG concentrations.

### 2.3. Gene Ontology (GO) Enrichment Analysis in Root and Shoot Tissues Across Different PEG Concentrations

#### 2.3.1. GO Enrichment Analysis in Root

The GO enrichment analysis delineated a pattern of specificity in the DEGs associated with varying PEG concentrations. In the roots, the downregulated genes exhibited a concentration-dependent enrichment in biological processes (Figure S1). At the lowest concentration of 1% PEG, the genes were notably involved in extracellular functions and responses to various abiotic stresses, including hydrogen peroxide, salt, and heat. As the PEG concentration escalated to 5%, the genes were enriched in a broader spectrum of stress responses, including those to heat, salt, hydrogen peroxide, general stress, abscisic acid, and ethylene signaling, along with specific molecular functions such as glutathione transferase activity and metabolic processes. At the highest concentration of 10% PEG, the enrichment shifted towards defense responses and signaling pathways, including those mediated by abscisic acid and ethylene, as well as processes related to the biosynthesis of green leaf volatiles and responses to jasmonic acid.

**Figure 3:**
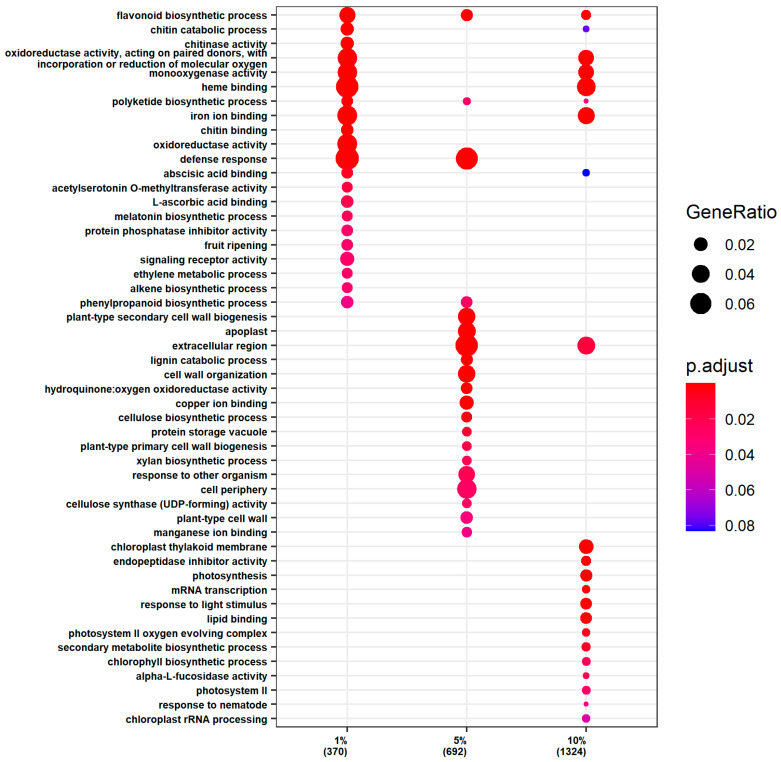
GO enrichment analysis of the upregulated genes in the roots.

Conversely, the upregulated genes in the roots showed a distinct pattern of enrichment with increasing PEG concentration (Figure 3). At 1% PEG, the genes were enriched in processes related to heme binding and defense mechanisms, including abscisic acid binding and the biosynthesis of secondary metabolites such as polyketides and flavonoids. At 5% PEG, the enrichment extended to cell wall organization and the biogenesis of plant secondary cell walls, alongside the continued enrichment in defense responses and secondary metabolite biosynthesis. At 10% PEG, the upregulated genes were involved in light-dependent processes, such as photosynthesis and chlorophyll biosynthesis, in addition to the previously mentioned pathways.

#### 2.3.2. GO Enrichment Analysis in Shoots

The GO enrichment analysis of DEGs in the shoot tissues has yielded a nuanced profile of enrichment that, while exhibiting specificity to the PEG concentration applied, also reveals some convergence with the root tissue response. The downregulation of gene expression in the shoot tissues was observed to be associated with distinct biological processes at each PEG concentration (Figure S2). At the lowest concentration of 1% PEG, the DEGs were notably enriched in pathways related to acetylserotonin O-methyltransferase activity and the melatonin biosynthetic process. As the PEG concentration increased to 5%, the enrichment profile of the downregulated genes shifted towards a broader spectrum of stress-related processes. These included heme binding, defense response, general stress response, heat response, iron ion binding, and glutathione transferase activity. At the highest PEG concentration of 10%, the downregulated genes were particularly enriched in defense response, ethylene-activated signaling pathway, response to jasmonic acid, and terpene synthase activity.

**Figure 4:**
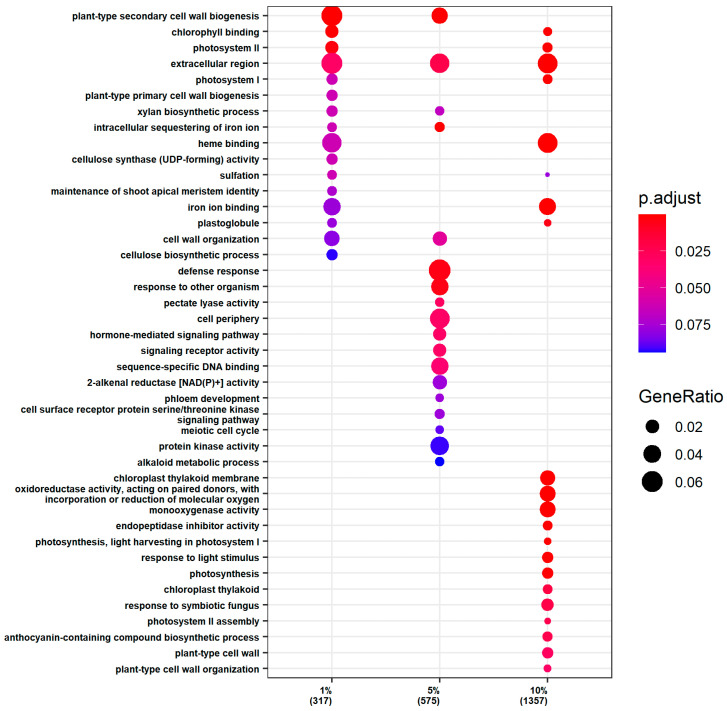
GO enrichment analysis of the upregulated genes in the shoots.

The upregulation of gene expression in the shoot tissues also displayed a concentration-dependent enrichment profile (Figure 4). At 1% PEG, the DEGs were enriched in processes associated with the extracellular region and plant-type secondary cell wall biogenesis, as well as in chlorophyll binding and photosystem II. At 5% PEG, the enrichment of upregulated DEGs extended to defense response and plant-type secondary cell wall biogenesis, suggesting a reinforcement of the cell wall and defense mechanisms to protect against the increasing stress. At 10% PEG, there was a pronounced enrichment in pathways related to photosynthesis and plant cell wall biosynthesis and organization. This included processes associated with the extracellular region, chlorophyll binding, photosystem I and II, heme binding, chloroplast thylakoid membrane, response to light stimulus, photosynthesis, photosystem II assembly, anthocyanin-containing compound biosynthetic process, plant-type cell wall, and plant-type cell wall organization.

A common thread across all PEG concentrations was the enrichment of DEGs in the extracellular region. Additionally, a shared enrichment in plant-type secondary cell wall biogenesis was observed at 1% and 5% PEG, while a convergence in chlorophyll binding and photosystem components was noted at 1% and 10% PEG.

### 2.4. GO-Based Pathway Analysis

#### 2.4.1. ABA Binding and Activated Signaling Pathway

The analysis of genes regulated by abscisic acid (ABA), a key hormone in drought stress response, revealed significant enrichment in pathways related to ABA binding, ABA-activated signaling pathway, and the regulation of the ABA-activated signaling pathway.

**Figure 5:**
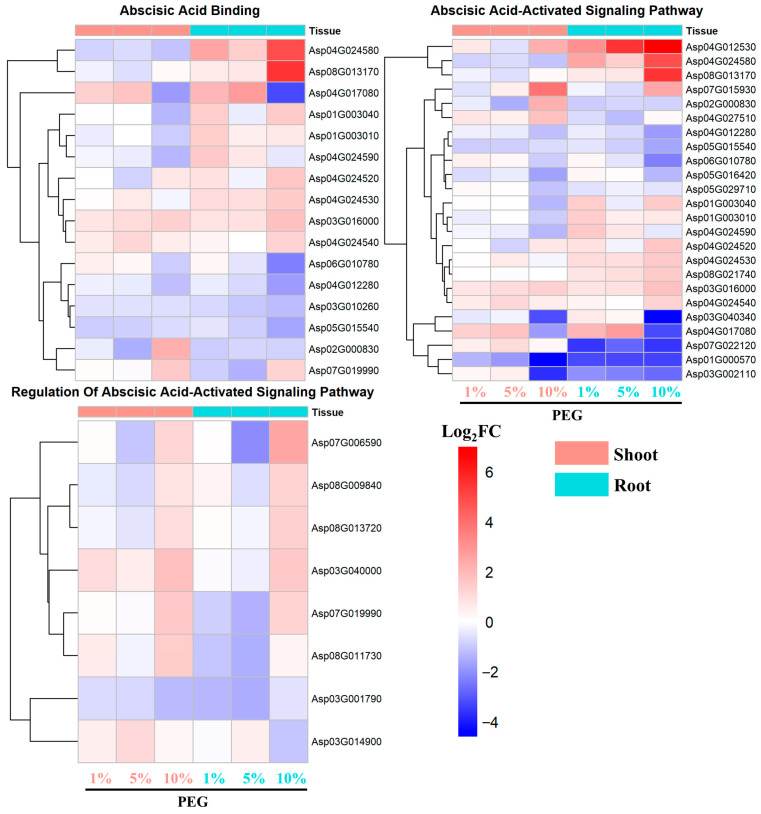
Expression pattern of DEGs involved in ABA binding and activated signaling pathway.

In the root tissues, the upregulation of genes involved in ABA binding was more pronounced and robust compared to the shoot tissues (Figure 5). Notably, genes such as Asp04G024580 and Asp08G013170 consistently showed upregulation across 1%, 5%, and 10% PEG treatments. Hierarchical clustering analysis grouped these genes together, indicating a coordinated response to osmotic stress. The expression fold changes (Log_2_FC) for Asp04G024580 and Asp08G013170 reached as high as 4.9 and 5.5, respectively.

Similar to the ABA binding genes, the root-specific genes in the ABA-activated signaling pathway exhibited a stronger and more consistent upregulation under PEG-induced stress (Figure 5). The genes including Asp04G024580, Asp04G012530, and Asp07G015930, demonstrated a significant increase in expression across all PEG concentrations. The Log_2_FC values for these genes peaked at 4.9, 5.5, and 7, respectively, underscoring their pivotal roles in the plant’s drought response.

The regulation of ABA-activated signaling pathway genes showed a distinct pattern, with upregulated genes predominantly induced under the 10% PEG condition, as compared to 1% and 5% PEG treatments (Figure 5). These genes were similarly induced in both root and shoot tissues, suggesting a systemic response to the highest osmotic stress level. The genes such as Asp07G006590, Asp08G009840, Asp08G013720, Asp03G040000, and Asp07G019990 exhibited a coordinated upregulation, indicative of a heightened regulatory response to severe drought conditions.

#### 2.4.2. Secondary Metabolite Pathways

Analysis of differentially expressed genes (DEGs) followed by Gene Ontology (GO) enrichment analysis revealed several enriched signaling pathways related to secondary metabolite production, such as the anthocyanin-containing compound biosynthetic process, diterpenoid biosynthetic process, melatonin biosynthetic process, flavonoid biosynthetic process, and polyketide biosynthetic process, in response to drought or osmotic stress.

**Figure 6:**
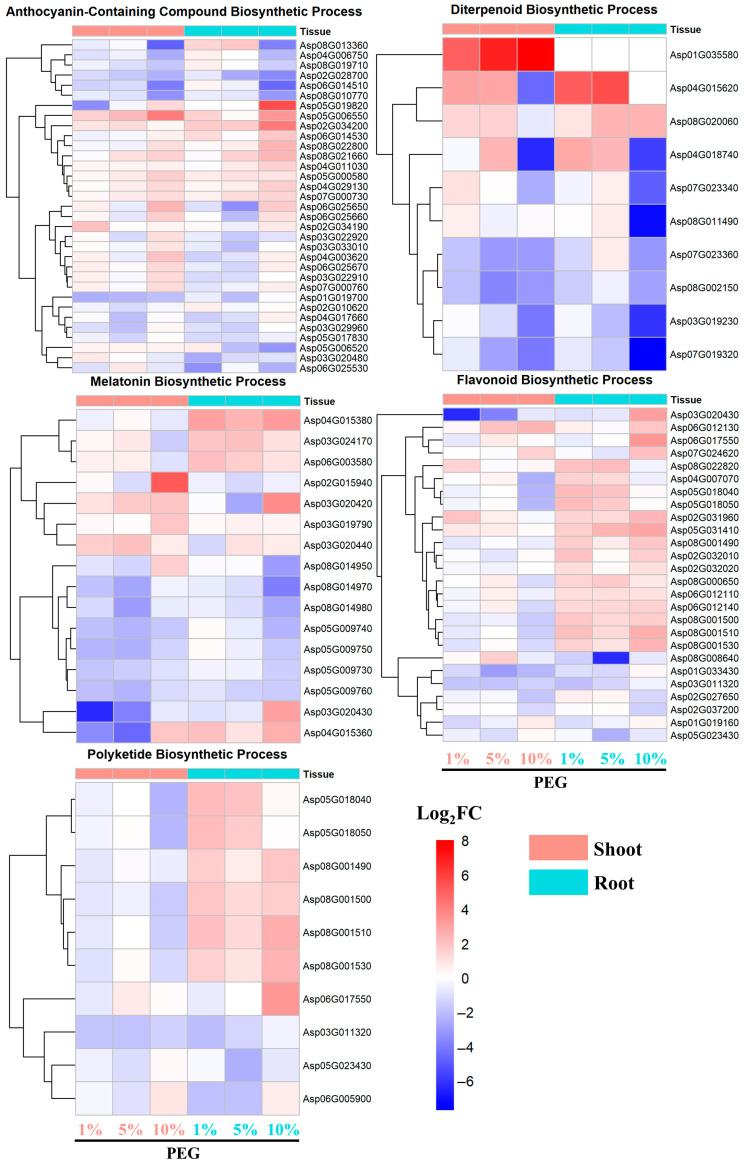
Expression pattern of DEGs involved in secondary metabolite biosynthesis pathway.

The upregulated genes involved in the biosynthesis of anthocyanin-containing compounds were induced in both root and shoot tissues under 1%, 5%, and 10% PEG conditions, with the most pronounced induction observed under 10% PEG (Figure 6). Notably, genes such as Asp05G019820 and Asp02G034200 in the root, and Asp05G006550 in the shoot, exhibited the highest Log_2_FC values of 5.6, 4.3, and 4.1, respectively.

In comparison, upregulated genes associated with the diterpenoid biosynthetic process displayed a differential expression pattern between root and shoot tissues (Figure 6). For instance, Asp01G35580 in the shoot was significantly upregulated with a Log_2_FC of 8 under 10% PEG, whereas Asp04G015620 was upregulated in both tissues, with a higher induction in the root, reaching a Log_2_FC of 5.6 under 5% PEG.

The genes involved in melatonin biosynthesis showed a stronger upregulation in the root tissue, with genes like Asp04G015380, Asp03G024170, and Asp06G003580 exhibiting Log_2_FC values of 3.2, 2.0, and 2.1, respectively, indicating a more robust response in the root than in the shoot under osmotic stress.

Likewise, the upregulation of genes in the flavonoid biosynthetic process was predominantly observed in the root tissue, with a noticeable increase in expression under all PEG concentrations, peaking under 10% PEG induction. The most significant upregulation was observed for Asp06G017550, with a Log_2_FC of 3.3. Similar to the flavonoid biosynthetic process, genes in the polyketide biosynthetic pathway were predominantly upregulated in the root tissue, with increasing expression levels as PEG concentration increased. The highest Log_2_FC values were observed for Asp06G017550, Asp08G001530, and Asp08G001510, with values of 3.3, 2.5, and 2.7, respectively.

#### 2.4.3. Photosynthesis-Related Pathways

**Figure 7:**
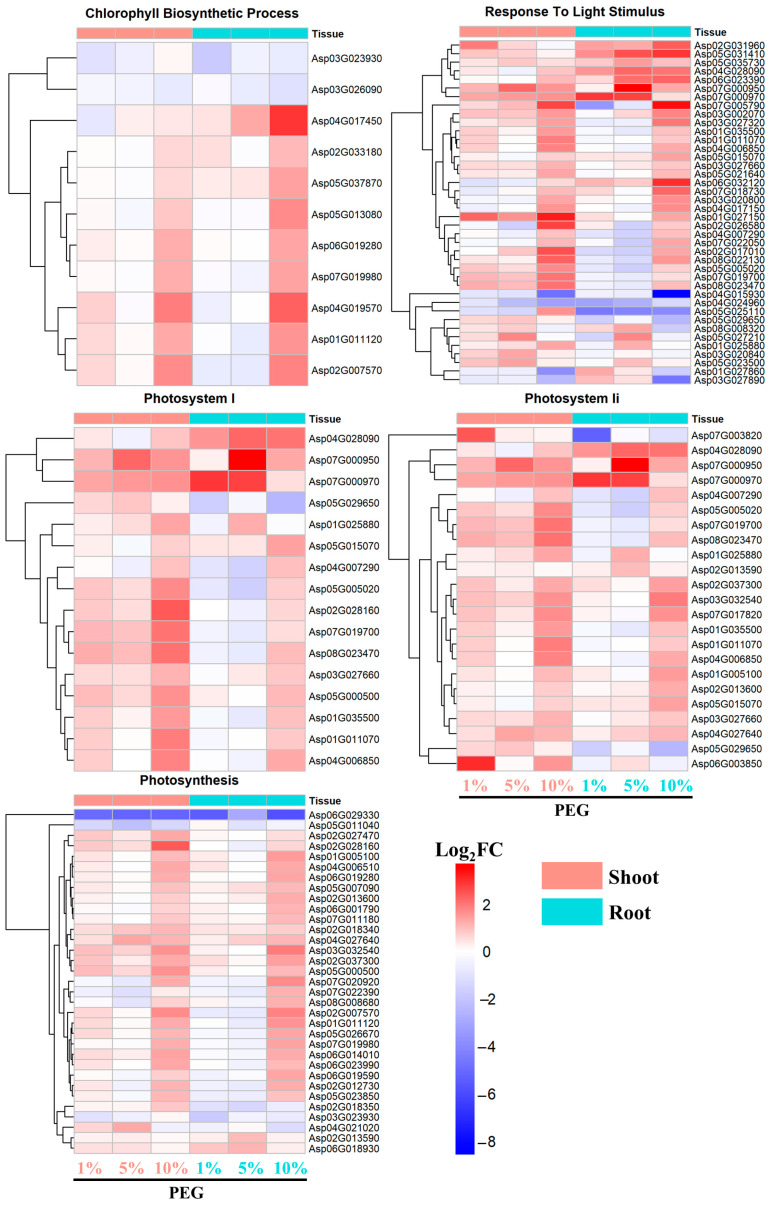
Expression pattern of DEGs involved in photosynthesis-related pathways.

As for the chlorophyll biosynthetic process, the majority of genes displayed an upregulated expression pattern in both root and shoot tissues. Notably, with the exception of a few genes such as Asp03G023930 and Asp03G026090, the upregulation of these genes was most pronounced under 10% PEG induction (Figure 7).

A similar trend was observed for genes associated with the response to light stimulus, where most genes showed the highest upregulation under 10% PEG induction. The genes Asp07G000950 and Asp07G005790 exhibited the highest Log_2_FC values of 3.7 and 3.4, respectively, indicating a significant response to high osmotic stress (Figure 7).

For genes related to photosystem I, photosystem II, and photosynthesis, a consistent pattern of upregulation was observed across all PEG concentrations. However, the intensity of upregulation was significantly higher in the shoot tissues compared to the root tissues, suggesting a more pronounced photosynthetic response in the aerial parts of the plant.

#### 2.4.4. Cell Wall Biogenesis and Organization

**Figure 8:**
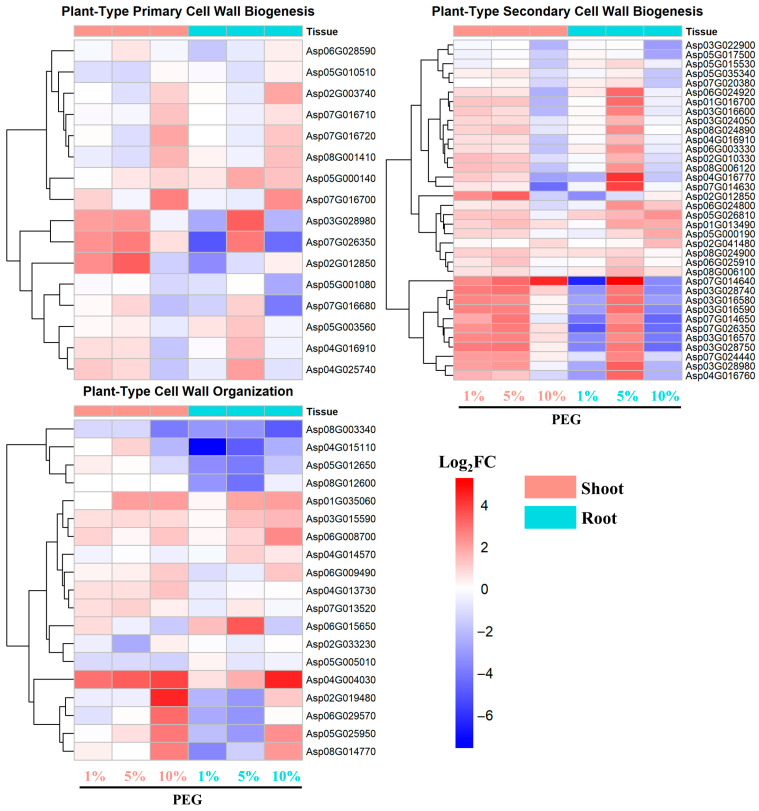
Expression pattern of DEGs involved in cell wall biogenesis and organization.

The majority of genes associated with plant-type primary cell wall biogenesis exhibited similar expression patterns in both root and shoot tissues, with a general trend of upregulation (Figure 8). Some genes showed the highest upregulation under 5% PEG induction, while others peaked under 10% PEG induction, with the highest Log_2_FC values around 3.

For genes involved in plant-type secondary cell wall biogenesis, there was an initial upregulation under 1% PEG stress, particularly in the shoot tissues, which showed a higher induction of expression compared to the root tissues. However, as the PEG concentration increased, the expression of these genes was suppressed, indicating a concentration-dependent regulatory pattern.

Genes related to plant-type cell wall organization responded similarly in both root and shoot tissues. Their expression levels increased with the increase in PEG concentration, reaching the highest levels under 10% PEG stress. Notably, genes such as Asp04G004030 and Asp02G019480 showed the most significant upregulation, with Log_2_FC values approaching 4, suggesting a critical role in the plant’s structural adaptation to osmotic stress (Figure 8).

#### 2.4.5. Signaling Pathways Responsive to Multiple Hormones

In our investigation into the complex hormonal interplay under PEG-mediated osmotic stress, we have uncovered a multifaceted response involving not only abscisic acid (ABA) but also jasmonic acid (JA), salicylic acid (SA), and gibberellin (GA) signaling pathways. Our findings, depicted in Figure 5 and Figure S3, reveal a coordinated upregulation of genes associated with these phytohormones, underscoring their integral roles in the plant’s adaptive mechanisms to osmotic stress. The gene expression patterns observed in both root and shoot tissues were consistent across the three tested PEG concentrations, demonstrating a biphasic response with approximately half of the genes upregulated and the other half downregulated in response to PEG treatment.

Among the upregulated genes, a concentration-dependent response to PEG was evident. For instance, Asp01G041030, encoding an ethylene-responsive transcription factor involved in the JA signaling pathway, exhibited a significant 5-fold upregulation exclusively under 10% PEG treatment, with minimal changes at lower concentrations. Similarly, Asp06G011030, encoding a WRKY domain-containing protein also responsive to JA, peaked at 2.3-fold upregulation under 10% PEG, highlighting the sensitivity of these genes to higher stress levels. In the SA signaling pathway, Asp06G002270, an ethylene-responsive transcription factor, reached its maximum induction of 5-fold under 10% PEG, with Asp05G021340, encoding a BTB/POZ and TAZ domain-containing protein, showing a 5.7-fold upregulation under the same conditions but remaining unresponsive at lower concentrations (Figure S3).

The gibberellin category mirrored this pattern, with Asp06G009540, encoding a glutathione transferase regulated by GA, exhibiting a remarkable 24-fold increase under 10% PEG treatment. Furthermore, Asp08G002120, encoding a GRAS transcription factor central to GA signaling, displayed PEG concentration-dependent upregulation, with a root-specific upregulation of up to 3.5 times (Figure S3). These findings underscore the intricate hormonal crosstalk and the dynamic gene expression patterns that plants employ to navigate PEG-induced osmotic stress, offering valuable insights into the molecular underpinnings of plant stress tolerance.

### 2.5. Hierarchical Clustering Analysis of Transcription Factors and Genes Involved in Osmotic Stress

#### 2.5.1. Identification of Differentially Expressed Transcription Factors (DETFs)

**Figure 9:**
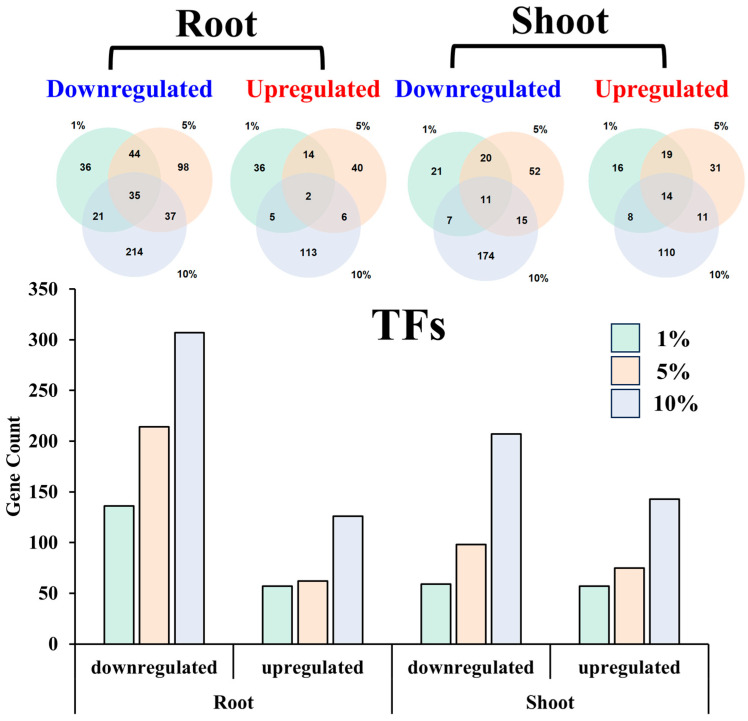
Differentially expressed transcription factors in root and shoot tissues across different PEG concentrations.

Transcription factors (DETFs) are pivotal in orchestrating the plant’s response to environmental stress. The differential expression analysis revealed a notable trend in the number of DETFs in response to the PEG treatments. Across both root and shoot tissues, the quantity of both downregulated and upregulated DETFs increased significantly with the concentration of PEG. Notably, the total number of downregulated DETFs substantially exceeded that of the upregulated ones, with this disparity being more pronounced in the root tissue (Figure 9).

In the root tissue, the number of downregulated DETFs escalated from 136 under 1% PEG treatment to 307 under 10% PEG treatment. Conversely, the number of upregulated DETFs in the root tissue increased from 57 under 1% PEG to 126 under 10% PEG. In the shoot tissue, the number of downregulated DETFs rose from 59 under 1% PEG to 207 under 10% PEG treatment, while the upregulated DETFs increased from 57 under 1% PEG to 143 under 10% PEG.

The comparative analysis of DETFs across different PEG concentrations within the same tissue revealed both shared and unique responses. For instance, in the root tissue, there were 35 downregulated DETFs common to all PEG concentrations, with each treatment also exhibiting a unique set of 36, 98, and 214 DETFs under 1%, 5%, and 10% PEG, respectively. Similarly, in the upregulated DETFs of the root, only two genes were common across all treatments, with each treatment having a unique set of 36, 41, and 113 DETFs.

In the shoot tissue, the downregulated DETFs shared among the three PEG concentrations were 11, while the unique sets for each treatment were not specified. For the upregulated DETFs in the shoot, the commonality was limited to 14 genes, with unique sets of 16, 31, and 110 DETFs under 1%, 5%, and 10% PEG, respectively.

These findings underscore the complexity of the transcriptional response in *A. camelorum* to drought stress, with a clear gradation in the expression of DETFs as a function of PEG concentration. The distinct patterns of DETFs in root and shoot tissues suggest tissue-specific regulatory mechanisms that contribute to the plant’s overall drought resistance strategy.

#### 2.5.2. Coexpression Patterns of Transcription Factors and Genes Involved in Osmotic Stress

**Figure 10:**
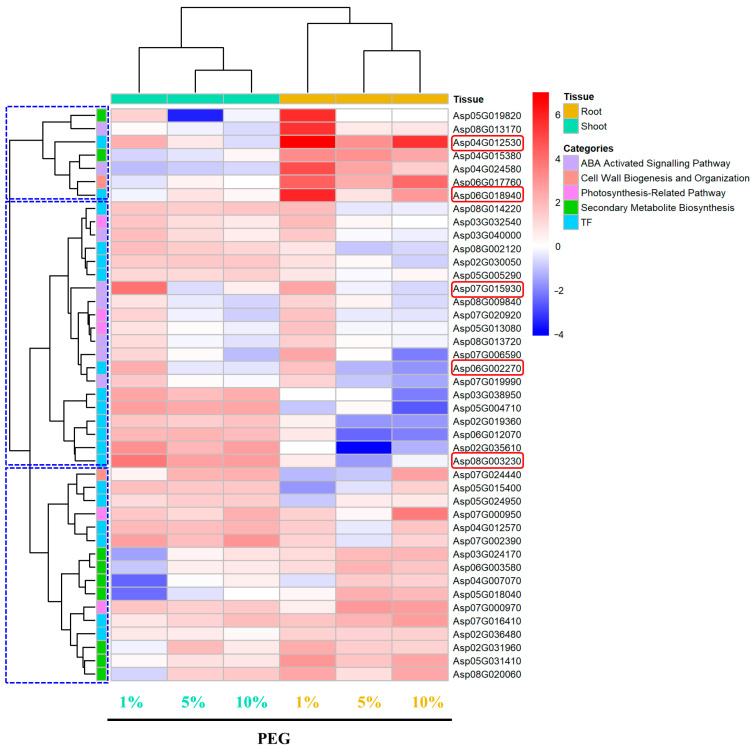
Hierarchical clustering analysis of TFs and selected genes (red box) involved in PEG-induced osmotic stress.

Overall, the expression patterns of selected genes under osmotic stress were categorized into six groups: ABA-activated signaling pathway, cell wall biogenesis and organization, PEG concentration-dependent and photosynthesis-related transcription factors (TFs), and secondary metabolite biosynthesis. The gene expression patterns were distinctly different between shoot and root tissues. The most notable gene, Asp01G030840, encoding a proline-rich protein, showed significant upregulation in both root and shoot tissues. Its expression levels increased linearly with PEG concentration (R^2^ = 0.99, Slope = 0.74, Table S2), reaching a log_2_FC of 12.5 in root tissues (Figure 10). Similarly, other linearly dependent genes such as Asp02G039780 (R^2^ = 0.82, Slope = 0.54), Asp03G006040 (R^2^ = 0.79, Slope = 0.50), and Asp03G008930 (R2 = 0.79, Slope = 0.50, Table S2) showed maximum log_2_FC values of 7.8, 7, and 4.3 in the shoot, respectively (Figure 10). These genes encode a BURP domain-containing protein, a non-specific lipid-transfer protein 5, and a pectinesterase inhibitor domain-containing protein. Additionally, PEG-concentration dependent genes prominently upregulated in shoot tissues included Asp01G035580 (R^2^ = 0.96, Slope = 0.32), encoding myrcene synthase, with a log_2_FC of 8, and Asp03G031750 (R^2^ = 0.71, Slope = 0.26, Table S2), encoding isoflavone 2′-hydroxylase, with a log_2_FC of 6.8. Another shoot-specific gene, Asp06G017370 (R^2^ = 0.92, Slope = 0.46, Table S2), encoding a light-regulated protein, showed a log_2_FC of 5.9.

In the ABA-activated signaling pathway, Asp04G012530, encoding an ethylene-responsive transcription factor ABI4, showed increased expression with PEG concentration, reaching a log_2_FC of 7. Asp04G017080, encoding an abscisic acid receptor PYL6, showed log_2_FCs of 2 and 2.8 at 1% and 5% PEG, respectively, but was downregulated to −3.2 at 10% PEG. For cell wall biogenesis and organization, Asp02G012850 and Asp07G026350, encoding cellulose synthase, had the highest log_2_FC of 3.3 and 2.7 in shoot tissues at 5% PEG. Asp02G019480 and Asp04G004030, encoding expansins, showed log_2_FC of 4.4 and 3.8 in shoot tissues under 10% PEG treatment (Figure 10). In the photosynthesis-related category, Asp04G028090, encoding a chlorophyll a-b binding protein, was notably upregulated in root tissues with a log_2_FC of 2.2 at 5% PEG. Shoot-specific upregulation was seen in Asp07G003820 and Asp06G003850, encoding photosystem II protein D1, with log_2_FC of 2.4 and 3, respectively. Likewise, Asp02G028160, Asp02G017010, and Asp08G022130, encoding photosystem I reaction center subunit N and protein LIGHT-DEPENDENT SHORT HYPOCOTYL 6, had log_2_FC of 2.4, 2.5, and 2.3 in the shoot, respectively. In secondary metabolite biosynthesis, Asp03G028190 and Asp04G015620, which encode beta-amyrin 28-monooxygenase isoform B and linalool synthase, showed the highest log_2_FC of 4.6 and 5.6 in roots.

Among transcription factors, Asp07G016410, encoding transcription activator-like effectors (TALE), had a highest log_2_FC of 2.7. It was closely clustered with a group of secondary metabolite biosynthesis genes such as Asp02G031960 (3-ketoacyl-CoA synthase) and a photosynthesis-related gene Asp04G028090, encoding a chlorophyll a-b binding protein (Figure 10). In contrast, Asp05G004710, encoding the transcription factor PIF7, was upregulated in root tissues with a log_2_FC of 2.7. It was clustered with Asp02G019360 (Transcription factor MYB61), Asp06G012070 (a GRAS transcription factor involved in gibberellin signaling), Asp07G002390 (AP2/ERF domain-containing transcription factor), and several photosynthesis-related genes, including Asp08G003230. Notably, Asp08G003230, encoding the C-repeat/dehydration-responsive element-binding factor 2 (ERF), was in the same cluster and had the highest log_2_FC of 3.8 in root tissues. This suggests a coordinated regulation of these pathways.

## 3. Discussion

### 3.1. Tissue-Specific and Concentration-Dependent Regulation of Osmotic Stress Response Pathways in A. camelorum

The primary objective of this study was to design a comprehensive experimental approach that integrates physiological and transcriptomic analysis to assess *A. camelorum*’s response to controlled osmotic stress. By subjecting the plant to a range of PEG concentrations, we aimed to mimic the progressive osmotic stress encountered during water deficit conditions and to study the tissue-specific and dose-dependent responses of the plant’s molecular and physiological reactions to drought. The use of PEG at varying concentrations to simulate drought stress revealed a significant inhibition of plant growth and pronounced phenotypic changes (Figure 1), which is consistent with the known effects of osmotic stress on plant physiology [20]. Our subsequent examination of the complex tissue-specific and concentration-dependent regulatory mechanisms through transcriptomic analysis has uncovered a number of key pathways categorized under GO terms that *A. camelorum* utilizes to counteract osmotic stress. These include the initiation of ABA signaling, the enhancement of secondary metabolite production, the adjustment of photosynthesis-related processes, and the elevation of cell wall biogenesis in a tissue-specific or concentration-dependent manner.

One of the main findings is that the root tissues of *A. camelorum* exhibit a more pronounced and robust upregulation of genes involved in ABA binding compared to the shoot tissues (Figure 5). The ABA signaling pathway is a well-known regulator of plant responses to water stress, modulating stomatal aperture and promoting the synthesis of stress-responsive proteins [21,22]. The activation of these pathways in *A. camelorum* may therefore be crucial for its drought resistance. This suggests that the root may play a central role in the initial perception and response to water deficit, aligning with the established role of ABA as a key hormone in drought stress signaling [23]. Furthermore, the ABA-activated signaling pathway genes in the root showed a distinct pattern of upregulation, predominantly under the 10% PEG condition, indicating a possible threshold effect where a certain level of stress is required to trigger a full response. Notably, the genes Asp04G024580 and Asp08G013170, encoding Major allergen Pru ar 1 and Major latex protein domain-containing protein, respectively, showed significant upregulation with Log_2_FC values of 4.9 and 5.5 in root induced by 10% PEG (Figure 5). These genes belong to the Major latex-like protein (MLP) family, which is known to be stress-responsive in many dicots, particularly towards drought, salt stress, plant hormones, and pathogen infections [24]. The homologs of these proteins, such as MLP43 in *Arabidopsis thaliana*, function as positive regulators during ABA responses and contribute to drought tolerance by modulating water loss efficiency, electrolyte leakage, ROS levels, and ABA-responsive gene expression [25]. MLPs can transport hydrophobic compounds over long distances through vascular bundles, which is essential for drought and salt tolerance through the mediation of plant hormone signaling pathways [24]. The concentration-dependent response observed in our study provides valuable insights into the dynamic nature of stress signaling and the potential for fine-tuning stress responses based on the severity of the stressor.

Our second findings reveal a significant PEG-concentration-dependent or tissue-dependent upregulation of genes involved in the biosynthesis of anthocyanin-containing polyketides, flavonoids, and diterpenoids in both root and shoot tissues, suggesting a strategic response to enhance stress tolerance through the production of protective compounds (Figure 6). This aligns with the known richness of secondary compounds in *A. camelorum*, including terpenes and flavonoids, which are crucial for mitigating oxidative stress and providing structural support [26,27]. Anthocyanins are known to have antioxidant properties and can protect plants from oxidative damage caused by stress [28]. The most pronounced induction under 10% PEG indicates that higher stress levels may trigger a more significant protective response. In comparison, the upregulation of genes in the flavonoid biosynthetic process and polyketide biosynthetic pathway was predominantly observed in the root tissue, with expression increasing with PEG concentration [29,30]. This suggests that roots may be the primary site for the production of these secondary metabolites, which can have roles in stress protection and signaling. The gene Asp05G019820 in the category of anthocyanin-containing compound biosynthetic process, encoding glycosyltransferase, stands out with a Log_2_FC value of 5.6 in roots induced by 10% PEG (Figure 6). Its homologs in *A. thaliana* contribute to stress tolerance by modulating anthocyanin accumulation and enhancing antioxidant activity [31], which underscores the importance of glycosyltransferases in the stress response of *A. camelorum*. Furthermore, the upregulation of genes Asp01G035580 and Asp04G015620, encoding myrcene and linalool synthase, respectively, in the diterpenoid biosynthetic process, indicates the involvement of these compounds in stress mitigation. Diterpenoids, known for their antioxidant properties, are crucial in avoiding drought-induced damage in plants [32]. The tissue and PEG-concentration dependence of Asp01G035580, and the PEG-concentration dependence of Asp04G015620, further emphasize the intricate regulatory network that *A. camelorum* employs to combat osmotic stress. In conclusion, our study provides insights into the complex regulatory mechanisms underlying the biosynthesis of secondary metabolites in *A. camelorum*. The tissue-specific and concentration-dependent upregulation of these genes not only underscores the multifaceted approach of *A. camelorum* to osmotic stress but also highlights the potential of these compounds in stress protection and signaling.

The photosynthesis-related pathways, including the chlorophyll biosynthetic process and response to light stimulus, showed an upregulated expression pattern in both tissues, with the highest induction under 10% PEG (Figure 7). For instance, the PEG-concentration-dependent expression of genes such as Asp04G017450, encoding spermidine sinapoyl-CoA acyltransferase, which is involved in spermidine biosynthesis, underscores the plant’s ability to enhance chlorophyll biosynthesis and photosynthesis under stress-induced conditions [33]. This is further supported by the upregulation of genes Asp04G019570 and Asp02G007570, encoding divinyl chlorophyllide 8-vinyl-reductase and magnesium chelatase, respectively, which are directly involved in chlorophyll biosynthesis and are activated under high PEG concentration (10%) conditions. These findings are intriguing as they contrast with the general trend of decreased photosynthesis and chlorophyll content under drought or osmotic stress [34]. Instead, *A. camelorum* appears to counteract these decreases by upregulating genes related to photosynthesis and chlorophyll, thereby maintaining its photosynthetic capacity [35,36]. Furthermore, the significant upregulation of genes encoding for chlorophyll a-b binding proteins (Asp07G000950, Asp07G000970), Photosystem I reaction center subunit N (Asp02G028160), and Photosystem II core complex proteins psbY (Asp03G032540), among others, under high PEG stress (Figure 7), suggests a coordinated response to maintain the functionality of the photosynthetic apparatus. This coordinated upregulation may be a key mechanism by which *A. camelorum* resists the detrimental effects of osmotic stress on photosynthesis, allowing the plant to continue energy production and carbon fixation, which are essential for survival and growth under water-limited conditions.

Lastly, the higher induction of genes related to cell wall biogenesis in the shoot tissue compared to the root tissues under PEG stress (Figure 8) indicates a possible role in modulating cell wall properties to withstand mechanical stress and maintain tissue integrity [37]. For example, the enhanced expression of cellulose synthase-encoding genes, such as Asp03G028980, Asp07G026350, and Asp02G012850, involved in primary cell wall biogenesis, in the shoot under low PEG concentrations indicates a heightened sensitivity of the shoot to osmotic stress in terms of primary cell wall regulation. This is further supported by the activation of genes encoding Fasciclin-like arabinogalactan proteins (Asp07G014640, Asp03G028740, Asp03G016580, Asp03G028750), which are crucial for secondary cell wall biogenesis and are known to be strongly induced under abiotic stresses [38], in the shoot at low PEG concentrations (1%), contrasting with their upregulation in the root only at higher concentrations (5%). This rapid response in the shoot suggests a prioritization of cell wall maintenance in the aerial parts of the plant, which are more directly exposed to the effects of drought [39]. Typically, plants under drought stress experience cell wall degradation in the shoot, leading to reduced growth, while maintaining root growth to ensure water uptake [40]. The observed increase in gene expression related to cell wall biogenesis in the shoot of *A. camelorum* may function as a compensatory mechanism to counteract cell wall degradation. This more sensitive upregulation in the shoot implies that under osmotic stress, cell wall degradation in the shoot occurs first, potentially to prioritize cell wall integrity in the root.

In conclusion, our study reveals a complex interplay between tissue-specific responses and concentration-dependent regulation of ABA signaling, secondary metabolites, photosynthesis efficiency, and cell wall biogenesis genes in *A. camelorum* under osmotic stress.

### 3.2. Multi-Hormonal Regulation of Osmotic Stress Responses in A. camelorum

The multi-hormonal regulation in *A. camelorum* under osmotic stress reveals a sophisticated network of interactions that underscores its remarkable drought resilience. The differential expression of genes linked to abscisic acid (ABA), jasmonic acid (JA), salicylic acid (SA), and gibberellic acid (GA) pathways in response to varying PEG concentrations highlights the plant’s intricate hormonal coordination. Our findings show that ABA, traditionally recognized as the primary hormone governing drought response, might collaborate with JA, SA, and GA pathways to create a dynamic regulatory framework. Notably, ethylene-responsive transcription factors (ERFs) such as Asp01G041030 (JA responsive pathwya) and Asp06G002270 (SA responsive pathway), significantly upregulated at 10% PEG (Figure S3), indicate their pivotal role in PEG-mediated osmotic stress. ERFs act as central hubs that mediate cross-talk between ABA, JA, and SA pathways, enhancing the plant’s adaptability to various biotic and abiotic stresses [41]. This regulatory function is consistent with observations in other species; for instance, the apple ERF, MdERF38, has been shown to promote anthocyanin biosynthesis under drought, contributing to stress tolerance [42]. The pronounced upregulation of Asp06G011030, a WRKY domain-containing protein under the JA-responsive GO category (Figure S3), further supports the role of WRKY transcription factors as crucial mediators that might link other hormone pathways. These proteins are reported to not only contribute to JA and SA signaling but also intersect with ABA pathways, exemplifying their multifaceted role in plants’ adaptation to stress conditions [43]. Similarly, the BTB/POZ and TAZ domain-containing protein, Asp05G021340, which showed a substantial upregulation by 10% PEG (Figure S3), is part of a protein family known to be involved in SA, JA, GA, and ABA signaling pathways [44]. The overexpression of such proteins in transgenic sweet potato has been shown to enhance drought tolerance by increasing proline biosynthesis and activating the ROS-scavenging system [45]. In addition, the upregulation of the GA signaling transcription factor, Asp08G002120, which belongs to the GRAS family, is particularly noteworthy (Figure S3). GRAS family proteins have been implicated in various stress and developmental processes in plants [46]. For instance, CaGRAS1 in *Capsicum annuum* acts as a positive regulator of the drought response, with the highest induction observed under drought, high salinity, and ABA treatments [47]. Thus, the complex multi-hormonal regulation observed in *A. camelorum* provides deeper insights into its genetic mechanisms for drought resistance, potentially informing the development of crops with enhanced stress tolerance.

### 3.3. Key Genes Involved in PEG-Induced Osmotic Stress Adaptation

Building on the insights from the tissue-specific and concentration-dependent regulation of osmotic stress response pathways in *A. camelorum*, and the multi-hormonal regulation of these responses that balance growth and adaptation, we now delve into the specific genetic elements that underpin the plant’s osmotic stress adaptation. By focusing on genes involved in significantly upregulated signaling pathways, as revealed by GO enrichment analysis, and those highly correlated with PEG concentration or significantly upregulated transcription factors (TFs, Figure 9), we have uncovered a set of key genes that underpin *A. camelorum*’s response to osmotic stress. Our objective is to contribute to the broader understanding of plant stress tolerance and to identify potential targets for genetic manipulation to enhance crop resilience in the face of water scarcity.

One of the most notable findings is the substantial dose-dependent upregulation of Asp01G030840 in both root and shoot tissues, with an R^2^ of 0.99 (Table S2), indicating a strong correlation with PEG concentrations. This gene encodes a proline-rich protein (Figure 10). This gene’s remarkable expression suggests its pivotal role in osmotic adaptation. Although the specific function of Asp01G030840 has not been previously described, proline-rich proteins (PRPs) are well-documented for their roles in abiotic stress resistance. For instance, the proline-rich protein MdPRP6 in apple has been shown to confer salt tolerance by preserving chloroplast structure, reducing reactive oxygen species (ROS) accumulation, and preventing Na leakage [48]. Similarly, PRPs are implicated in various abiotic stresses, including drought, salt, heat, and cold, as well as in response to zinc stimuli, in a tissue-specific manner [49]. The multifunctional nature of PRPs includes the regulation of cellular proline levels in response to abiotic stress intensities and durations, aligning with our observation of the PEG-concentration-dependent expression of Asp01G030840 [50].

Another gene of interest is Asp02G039780, which encodes a BURP domain-containing protein (Figure 10). This gene also exhibits PEG-concentration-dependent expression with an R^2^ of 0.82 (Table S2), such strong correlation indicating the importance of BURP domain-containing proteins in osmotic and drought stress responses. All 39 BURP genes in *Medicago truncatula* were found to be regulated by drought stress [51], and differential expression of BURP genes was observed in *Phaseolus vulgaris* under drought conditions [52]. This suggests that BURP proteins play critical roles in drought stress adaptation. Additionally, the non-specific lipid-transfer protein Asp03G006040 was highly upregulated in response to increasing PEG concentrations. Members of these protein families are known to regulate ROS scavenging and reorganize lipid profiles to withstand osmotic and drought stress conditions [53].

The significant upregulation of genes associated with cell wall biogenesis and organization in response to PEG-induced osmotic stress highlights the critical role of cell wall dynamics in the adaptation of *A. camelorum*. Notably, the gene Asp03G008930, which encodes a Pectinesterase inhibitor domain-containing protein, demonstrated a strong linear correlation with PEG concentration (R^2^ = 0.93, Table S2), suggesting its pivotal involvement in pectin modification and the regulation of cell wall porosity. This modulation of cell wall properties likely enhances the plant’s ability to adapt to various abiotic stresses, aligning with findings by Hong et al. [54] that highlight the role of cell wall remodeling in stress tolerance. Intriguingly, the upregulation of a similar protein is observed exclusively in drought-stressed anthers of the drought-sensitive rice genotype IR64, as reported by Liu et al., 2011 [55], which may suggest a contrasting negative regulatory role in drought/osmotic stress response. Furthermore, the pronounced upregulation of the Fasciclin-like arabinogalactan protein Asp08G003230 (up to 21 times with increasing PEG concentrations, R^2^ of 0.98, Table S2) indicates the significance of cell wall restructuring in stress adaptation. These proteins, which are known for their functions in cell adhesion and signaling, may bolster the plant’s osmotic stress tolerance by modulating cell wall properties. This finding is corroborated by Chowdhury et al. [56], who identified a Fasciclin-like arabinogalactan protein as a principal drought-responsive gene through machine-learning analysis of diverse tomato genotypes, tissue types, and drought durations, thereby contributing to drought adaptation in tomato. The involvement of Fasciclin-like arabinogalactan proteins in a spectrum of other abiotic and biotic stresses, such as cold, salt, and pathogen challenges [57], further underscores their multifaceted role in plant stress response. Collectively, our findings not only advance the understanding of the genetic underpinnings of osmotic stress adaptation in *A. camelorum* but also highlight potential targets for genetic manipulation to enhance the resilience of crops to water scarcity and other environmental stressors.

The exploration of *A. camelorum*’s adaptive mechanisms to PEG-induced osmotic stress has led us to focus on key genes involved in secondary metabolite biosynthesis, which were found to be highly upregulated in a PEG-concentration-dependent manner. This observation is particularly significant, as it suggests a direct link between the plant’s metabolic responses and its ability to cope with osmotic stress. Notably, myrcene synthase (Asp01G035580, with an R^2^ of 0.96, Table S2) and isoflavone 2′-hydroxylase (Asp03G031750, with an R^2^ of 0.7, Table S2) were both significantly upregulated in roots (Figure 10), indicating their potential roles in the plant’s drought and osmotic stress response. Myrcene synthase, responsible for the biosynthesis of essential oils, is implicated in drought stress in plants. For instance, the tissue-specific expression of terpene synthase genes in *Rosa chinensis* under osmotic stress suggests a role in stress adaptation [58]. The production of monoterpenes in Scots pine needles under drought conditions further underlines the importance of these volatiles for stress tolerance [59]. This aligns with our findings, where myrcene synthase is upregulated in response to increasing PEG concentrations, potentially conferring antioxidative activity to mitigate reactive oxygen species (ROS) accumulation, as suggested by Zhang [60] and Nakabayashi [61]. Similarly, isoflavone 2′-hydroxylase, which is involved in flavonoid biosynthesis, plays a crucial role in drought and osmotic stress adaptation [62]. Flavonoids are known for their antioxidant properties and their ability to protect plants from oxidative damage under stress conditions. The upregulation of isoflavone 2′-hydroxylase in *A. camelorum* under osmotic stress suggests a similar protective role, potentially enhancing the plant’s tolerance to water deficit conditions.

Phytohormones and associated transcription factors regulate plant resistance responses to both biotic and abiotic stresses. In response to abiotic stresses, abscisic acid (ABA) is one of the most critical players. Not surprisingly, abscisic acid receptor PYL6 (Asp04G017080) and ethylene-responsive transcription factor ABI4 (Asp04G012530) implicated in ABA signaling were significantly upregulated (Figure 10). The expression of ABSCISIC ACID-INSENSITIVE (ABI) proteins in *Arabidopsis* is upregulated in the ABA signaling pathway, regulating downstream genes in drought stress responses [63]. Other transcription factors such as TALE (Asp07G016410), MYB61 (Asp02G019360), GRAS (Asp06G012070), and ERF (Asp07G002390 and Asp08G003230) were significantly upregulated and clustered with key secondary metabolite biosynthesis genes and chlorophyll a-b binding protein, suggesting their key regulatory function. For example, a *Citrus sinensis* MYB61 regulates stomata opening, stomatal conductance, and respiration rate in a tissue-specific manner, thereby improving water-use efficiency and drought stress tolerance in Arabidopsis [64]. Besides, ERFs are a class of transcription factors involved in various abiotic stress conditions and interact with various phytohormones such as ABA, ethylene, gibberellin, and auxin [65]. Notably, a GRAS transcription factor involved in gibberellin signaling in our study was also markedly upregulated, suggesting a potential role in osmotic adaptation. One GRAS transcription factor, BrLAS, from *Brassica rapa* plays a role in both development and abiotic stress by promoting cell elongation during root development and enhancing antioxidant enzyme and ROS scavenging activity under drought treatment [66].

Taken together, our stud identified several key genes crucial for osmotic stress adaptation in *A. camelorum*, with notable upregulation in signaling pathways, transcription factors, and genes involved in cell wall biogenesis and secondary metabolite biosynthesis. The significant expression of genes such as the proline-rich protein, BURP domain-containing proteins, and various transcription factors highlights their potential roles in enhancing osmotic stress resistance through diverse molecular mechanisms and tissue-specific responses.

## 4. Materials and Methods

### 4.1. Plant Material and PEG Treatments

*A. camelorum* seeds were collected from mature plants growing in natural habitats in arid regions. Seeds were surface-sterilized and germinated in MS medium with different PEG concentrations (0%, 1%, 5%, 10%) under controlled environmental conditions, including a 16-h photoperiod, temperature of 25 °C, and relative humidity of 60%. The medium pH was adjusted to 5.8.

### 4.2. Phenotypic Analysis

Morphological parameters, including plant height, internode length, and root length, were measured before and after PEG treatment at regular intervals. Plant height was measured from the medium surface to the tip of the longest leaf. Internode length and root length were measured using a ruler.

### 4.3. Transcriptome Analysis

Total RNA was extracted from the stems and roots of treated and untreated plants using a commercial RNA extraction kit (TIANGEN BIOTECH, Beijing, China). RNA quality and quantity were assessed using a NanoDrop spectrophotometer (Thermo Fisher Scientific, Waltham, MA, USA) and agarose gel electrophoresis (Bio-Rad Laboratories, Hercules, CA, USA). RNA sequencing (RNA-Seq) libraries were prepared using the Illumina TruSeq Stranded mRNA Library Prep Kit (Illumina, Inc., New York, NY, USA) and sequenced on an Illumina HiSeq platform. The raw sequencing reads were subjected to quality control using cutadapt software v4.3 to remove adapter sequences and low-quality reads. This step was crucial for eliminating reads with a length below 50 nucleotides and those containing more than 10% ambiguous ‘N’ bases, as well as for the removal of ribosomal RNA (rRNA) sequences to enrich for mRNA sequences relevant to our study. The filtered reads were then aligned to the reference genome of *A. camelorum* using the STAR aligner v2.7.10b, which employs default mapping parameters to ensure accurate and sensitive read mapping.

### 4.4. Differential Gene Expression and Functional Enrichment Analysis

Gene expression levels were quantified as Fragments Per Kilobase of transcript per Million mapped reads (FPKM) using featureCounts software v2.0.4. The R package DESeq2 v1.44.0 was employed to perform differential gene expression analysis. Genes with a fold change ≥2 and a false discovery rate (FDR) < 0.05 were deemed differentially expressed. Venn diagrams were constructed using the R package VennDiagram v1.7.3 to illustrate the overlap of differentially expressed genes (DEGs) among the different PEG treatments.

Gene Ontology (GO) pathway enrichment analysis was conducted using the online webserver eggNOG-mapper (http://eggnog-mapper.embl.de/, accessed on 1 May 2024) to identify biological processes and pathways associated with the differentially expressed genes (DEGs). The enrichment analysis was performed and visualized using the R package clusterProfiler v4.6.2.

### 4.5. Identification of Key Pathways and Hierarchical Cluster Analysis

Key pathways were identified, and hierarchical cluster analysis was performed using the R package pheatmap v1.0.12 to visualize the gene expression heatmap, focusing on significantly enriched GO categories. This analysis aimed to identify transcription factors and drought-responsive genes that showed consistent upregulation across all PEG concentrations in either root or shoot tissues.

### 4.6. Clustered Coexpression Patterns of Transcription Factors and Drought-Responsive Genes

Transcription factors were identified using the online PlantTFDB webserver (https://planttfdb.gao-lab.org/, accessed on 5 May 2024). Hierarchical cluster analysis was used to perform co-expression analysis on selected genes, including transcription factors (TFs) consistently upregulated in response to PEG treatment. In addition to TFs, other genes identified through GO enrichment analysis were included, focusing on those involved in secondary metabolite biosynthesis, photosynthesis-related pathways, ABA-activated signaling pathways, and cell wall biogenesis and organization. Another category included genes that showed dose-dependent upregulation with PEG concentrations in either root or shoot.

## 5. Conclusions

The comprehensive analysis of *A. camelorum*’s response to osmotic stress induced by varying PEG concentrations has yielded significant conclusions regarding its drought resistance mechanisms. Our study demonstrates that under controlled laboratory conditions, *A. camelorum* exhibits progressive growth inhibition and phenotypic plasticity in response to increasing osmotic pressure, underpinned by a complex interplay of molecular and physiological adjustments. Transcriptomic profiling has illuminated the activation of key pathways, such as the ABA signaling cascade, cell wall dynamics, photosynthesis, and secondary metabolism, which are central to the plant’s adaptive strategies under drought conditions. The distinct tissue-specific and PEG concentration-dependent expression patterns of these pathways highlight the intricate regulatory networks at play in osmotic stress adaptation.

Particularly noteworthy are the upregulated genes encoding proline-rich proteins, BURP domain-containing proteins, cell wall-modifying enzymes, and secondary metabolite biosynthesis enzymes, which collectively contribute to the plant’s osmotic stress tolerance. The identification of transcription factors such as ABI4, TALE, MYB61, GRAS, and ERF, which are implicated in phytohormone signaling, further underscores the multilayered regulation of drought responses in *A. camelorum*. These findings provide a solid foundation for future research aimed at dissecting the functional roles and interactions of these key genes and regulatory elements, which are essential for advancing our understanding and management of drought tolerance in desert plants. The elucidation of these mechanisms not only broadens our knowledge of plant stress biology but also holds promise for the development of strategies to improve the resilience of crops to water-limited environments.

## Data Availability

The data generated and/or analyzed during the current study are available from the corresponding author upon reasonable request.

## Supplementary Materials

The following supporting information can be downloaded at https://www.mdpi.com/article/10.3390/ijms252312725/s1.
