# Supplementary material for: Exploring the Genetic Basis of Drought Tolerance in Alhagi camelorum: A Comprehensive Transcriptome Study of Osmotic Stress Adaptations
Source: Int J Mol Sci. 2024 Nov 27;25(23):12725. doi: 10.3390/ijms252312725 (PMC11641545; doi:10.3390/ijms252312725)
Supplement: Supplementary file 1 [file ijms-25-12725-s001.zip › Supplementary Figures.pdf]

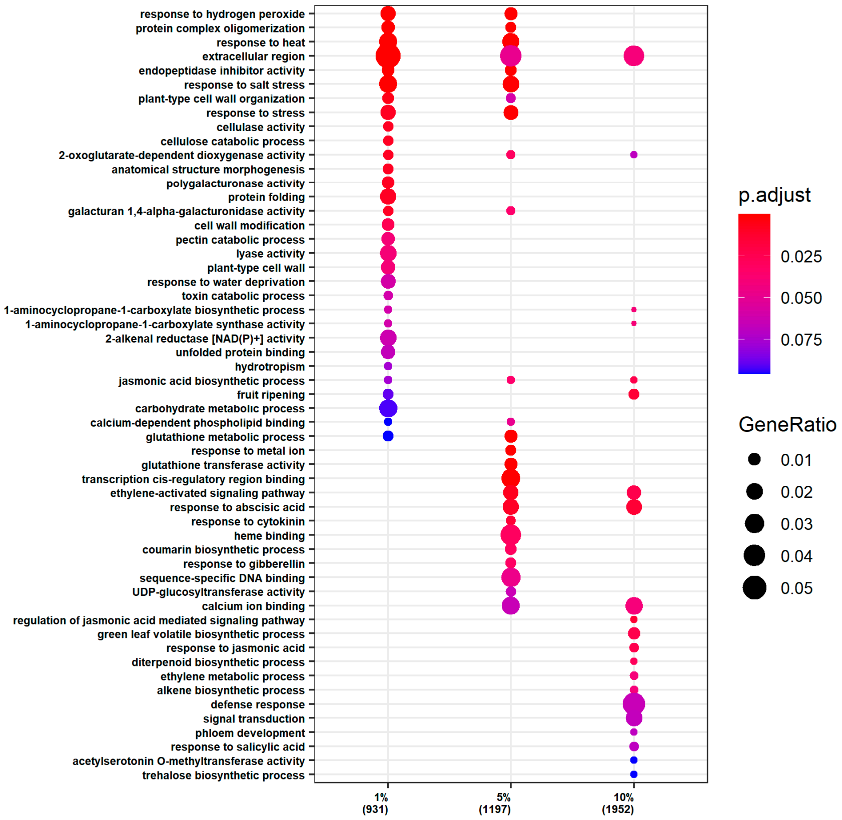

Figure S1. GO enrichment analysis of the downregulated genes in the roots

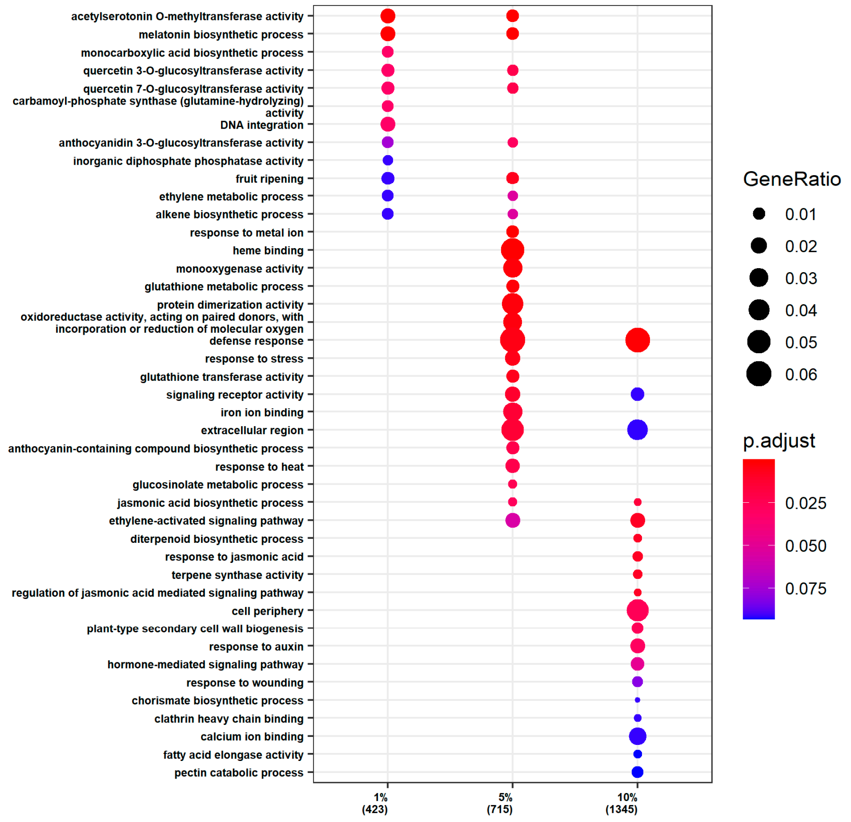

Figure S2. GO enrichment analysis of the downregulated genes in the shoots

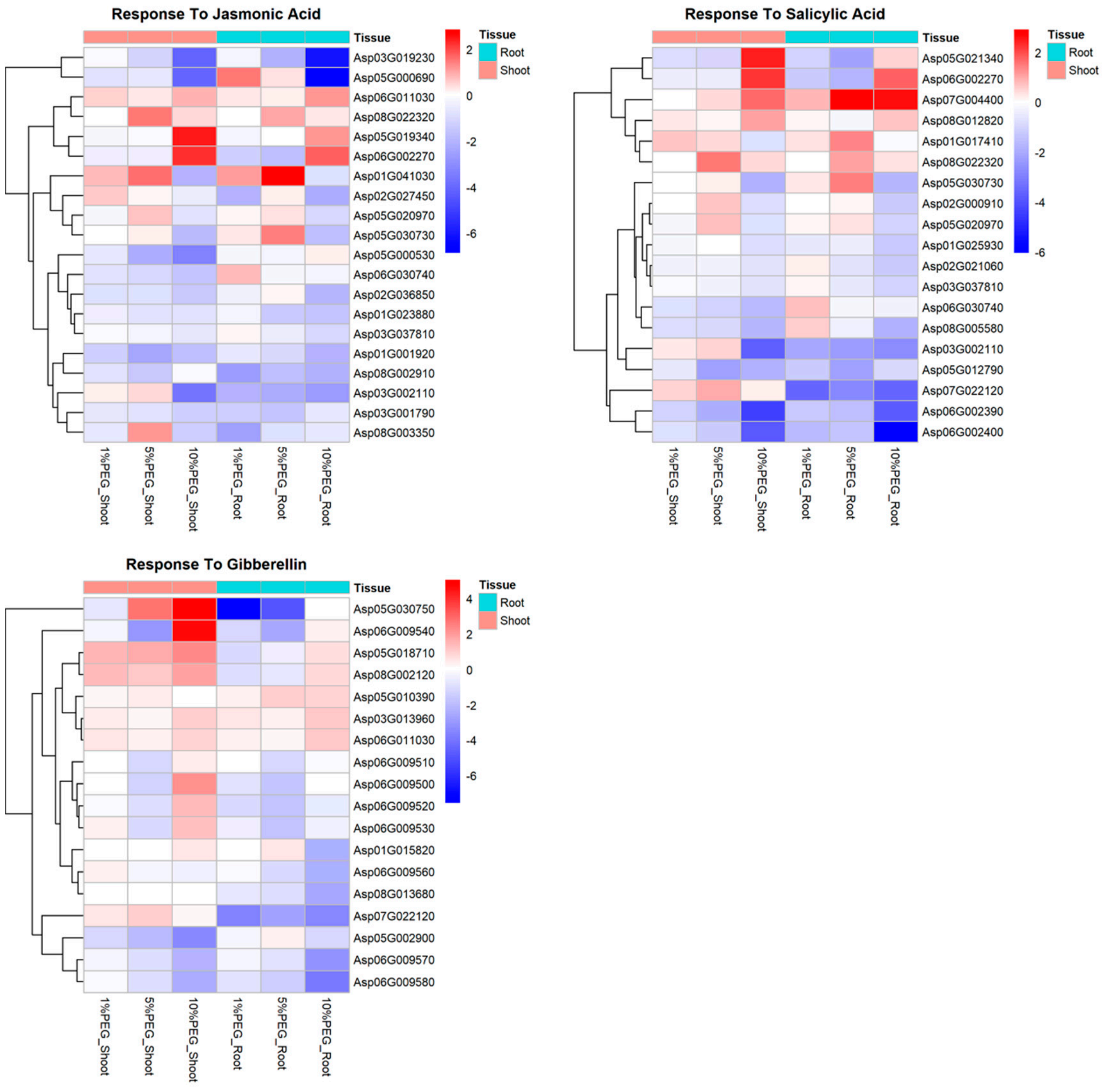

Figure S3. The expression of genes-related to Jasmonic Acid, Salicylic Acid and Gibberellin in both root and shoot tissues to different PEG treatments
